# Papain-Like Cysteine Protease Gene Family in Fig (*Ficus carica* L.): Genome-Wide Analysis and Expression Patterns

**DOI:** 10.3389/fpls.2021.681801

**Published:** 2021-05-28

**Authors:** Yanlei Zhai, Yuanyuan Cui, Miaoyu Song, Alexander Vainstein, Shangwu Chen, Huiqin Ma

**Affiliations:** ^1^College of Horticulture, China Agricultural University, Beijing, China; ^2^The Robert H. Smith Faculty of Agriculture, Food and Environment, The Hebrew University of Jerusalem, Rehovot, Israel; ^3^College of Food Science and Nutritional Engineering, China Agricultural University, Beijing, China

**Keywords:** papain-like cysteine protease, gene architecture, transcriptome, proteome, *Ficus carica* L.

## Abstract

The papain-like cysteine proteases (PLCPs) are the most abundant family of cysteine proteases in plants, with essential roles in biotic/abiotic stress responses, growth and senescence. Papain, bromelain and ficin are widely used in food, medicine and other industries. In this study, 31 PLCP genes (*FcPCLP*s) were identified in the fig (*Ficus carica* L.) genome by HMM search and manual screening, and assigned to one of nine subfamilies based on gene structure and conserved motifs. SAG12 and RD21 were the largest subfamilies with 10 and 7 members, respectively. The *FcPCLP*s ranged from 1,128 to 5,075 bp in length, containing 1–10 introns, and the coding sequence ranged from 624 to 1,518 bp, encoding 207–505 amino acids. Subcellular localization analysis indicated that 24, 2, and 5 PLCP proteins were targeted to the lysosome/vacuole, cytoplasm and extracellular matrix, respectively. Promoter (2,000 bp upstream) analysis of *FcPLCP*s revealed a high number of plant hormone and low temperature response elements. RNA-seq revealed differential expression of 17 *FcPLCP*s in the inflorescence and receptacle, and RD21 subfamily members were the major *PLCP*s expressed in the fruit; 16 and 5 *FcPLCP*s responded significantly to ethylene and light, respectively. Proteome analyses revealed 18 and 5 PLCPs in the fruit cell soluble proteome and fruit latex, respectively. Ficins were the major PLCP in fig fruit, with decreased abundance in inflorescences, but increased abundance in receptacles of commercial-ripe fruit. *FcRD21B/C* and *FcALP1* were aligned as the genes encoding the main ficin isoforms. Our study provides valuable multi-omics information on the FcPLCP family and lays the foundation for further functional studies.

## Introduction

Cysteine proteases, which contain a cysteine residue at their active catalytic site, catalyze the hydrolysis of peptides and proteins. Cysteine proteases can be divided into 11 clans with different evolutionary routes; different families in the same clan are diverse in sequence and structure (Bah et al., 2006; Rawlings et al., 2016). C1A is the largest family of the CA clan of papain-like cysteine proteases (PLCPs) (Rawlings et al., 2010). Members of the PLCP family in plants, such as papain, chymopapain, caricain (*Carica papaya*), bromelain (*Ananas comosus*) and ficin (*Ficus carica*), have broad substrate specificity and strong proteolytic activity, and the enzymes are of high commercial value in cheese making (Mazri et al., 2018), meat tenderization (Sullivan and Calkins, 2010; Bekhit et al., 2014), beer stabilization, biscuit baking and leather softening, as versatile biocatalysts (Huang et al., 2008) and in making digestive drugs (Zhalehjoo and Mostafaie, 2012).

In plants, PLCPs act as an immunity hub, playing critical roles in plant–pathogen/pest interactions and abiotic stress responses (Shindo and Van Der Hoorn, 2008; Misas-Villamil et al., 2016). PLCPs are first synthesized as inactive precursors with a signal peptide, an N-terminal self-inhibiting predomain, and a mature catalytic domain (Richau et al., 2012; Wang et al., 2014). The mature PLCPs are monomer proteases consisting of an α-helix and β-sheet domain of similar size, forming an active cleft that specifically binds with the substrate. The active catalytic site—the highly conserved catalytic triad Cys–His–Asn—is located at the cleft, which is the conserved characteristic of PLCPs (Polaina and MacCabe, 2007). Most PLCPs have small molecular masses, ranging from 20 to 35 kDa, and a few are 50–75 kDa. The optimum pH for catalytic activity is 5.0–8.0 (Dubey et al., 2007). In plants, PLCPs are divided into nine subfamilies according to the propeptide domain and characteristic motifs: RD21A-like, CEP1-like, XCP2-like, XBCP3-like, THI1-like, SAG12-like, RD19A-like, ALP-like, and CTB-like (Richau et al., 2012).

Fig (*Ficus carica* L.) is a latex-producing fruit crop; the milk-like latex flows out when fig tissue is wounded (Raskovic et al., 2016). The latex participates in defense against fungi and insects (Mnif et al., 2015) and has historically been used to treat skin diseases. On the other hand, the proteases in latex damage the skin of fig pickers and workers in the orchard, and in commercial-ripe fig fruit, the latex needs to be drained after harvesting (Flaishman et al., 2008).

PLCPs—dominated by ficin isoforms—are the major protein component of fig latex (Zare et al., 2013; Raskovic et al., 2014). Ficin (EC 3.4.22.3), also known as ficain, is widely found in *Ficus* species. Our previous study revealed that multiple cysteine proteases, i.e., ficins A, B, C, D, and cysteine protease RD21A, make up the large proportion of the fig fruit’s soluble proteome in both commercial-ripe and tree-ripe fruit, but transcripts of ficin isoforms were not identified by RNA-seq (Cui et al., 2019).

Publication of the fig (*F. carica*) genome (Usai et al., 2020) has provided the necessary information for bioinformatics analyses of the FcPLCP family. In the present study, gene structures, conserved motifs, phylogenetic relationships, and promoter *cis*-elements of *FcPLCP*s were analyzed, and gene-transcription patterns and protein abundance in fig fruit were revealed. This combined genomic, transcriptomic and proteomic study provides a matrix of information on the PLCP family in fig, laying a sound foundation for the identification of important PCLPs for further studies of biological function.

## Materials and Methods

### Identification of PLCP Genes From the Genome of *Ficus carica*

Genomic data of *F. carica* and *Morus notabilis* were downloaded from NCBI^1^, genomic data of *Arabidopsis thaliana* were downloaded from the TAIR database^2^, data of *Ficus hispida* and *Ficus microcarpa* were downloaded from the Genome Sequence Archive (GSA) and Genome Warehouse (GWH) database^3^. The gene and coding sequences (CDSs) were extracted from contig level sequences (BioProject: PRJNA565858^4^) using TBtools (Chen et al., 2020), according to gene location information in Usai et al. (2020). The protein sequences were translated based on the CDSs.

The Hidden Markov Model (HMM) file corresponding to the peptidase C1 domain (PF00112) was downloaded from the Pfam database (El-Gebali et al., 2019). HMMER was used to search for the PLCPs from *F. carica*, *A. thaliana*, *M. notabilis, F. hispida*, and *F. microcarpa* genome databases with default parameters. All candidate proteins were confirmed by Pfam and the conserved domain database (Marchler-Bauer and Bryant, 2004; NCBI CDD^5^). Redundant sequences, sequences with E-values less than 1E-20, and sequences with no peptidase C1 domain were eliminated.

### Multiple Sequence Alignment, and Phylogenetic and Sequence Feature Analyses

The PLCP sequences of *F. carica*, *A. thaliana*, *M. notabilis, F. hispida*, and *F. microcarpa* were subjected to multiple sequence alignment using ClustalW with default parameters. An unrooted phylogenetic tree based on this alignment was constructed using the neighbor-joining method by MEGA X, with the following parameters: Poisson model, pairwise deletion, 1,000 bootstrap replications. PLCPs were named according to their homology with *Arabidopsis*.

Sequence length, molecular weight, isoelectric point (pI), and the grand average of hydropathicity (GRAVY) index of PLCP proteins were computed by ProtParam^6^ (Gasteiger et al., 2005). The signal peptide and subcellular location were predicted by SignalP^7^ (Armenteros et al., 2019) and DeepLoc^8^ (Armenteros et al., 2017).

Exon–intron positions were obtained by genome annotation. The conserved motifs of PLCPs of *F. carica* were computed by the MEME program (Bailey et al., 2009), with the following parameters: classic mode; site distribution, zero or one occurrence per sequence; number of motifs, 20; width of motifs, between 6 and 50 residues. PROSITE (Sigrist et al., 2012) and NCBI CDD were used for motif analysis. The diagrams were drawn with TBtools (Chen et al., 2020).

### Chromosomal Location, Gene Duplication, and Promoter Analysis

Papain-like cysteine proteases genes were mapped to *F. carica* chromosomes using TBtools (Chen et al., 2020). The Multiple Collinearity Scan toolkit (MCScanX) was used to conduct the syntenic analysis among *F. carica*, *F. hispida*, and *F. microcarpa* (Wang et al., 2012).

An all-against-all BLASTP alignment was run to reveal potential gene duplication. The criteria for duplicated pairs were: (a) length of the aligned sequence covers >75% of the longer gene, and (b) >75% similarity of aligned regions (Vatansever et al., 2016). Corresponding coding regions were aligned using ClustalW. The number of non-synonymous substitutions per non-synonymous site (*K*_a_) and the number of synonymous substitutions per synonymous site (*K*_s_) were calculated by KaKs_Calculator 2.0 (Wang et al., 2010). The gene duplications were dated using the formula *T* = *K*_s_/2*r*; *r*, which is the rate of divergence for nuclear genes, was taken to be 7 × 10^–9^ synonymous substitutions per site per year according to a previous report in *A. thaliana* (Ossowski et al., 2010).

Promoter analysis was conducted using Plant CARE (Lescot et al., 2002), based on the 2000-bp sequence upstream of the gene.

### Plant Materials

The common fig cultivar Zibao, planted at the Shangzhuang Experimental Station of China Agricultural University (Peking, China), served as the plant material. Based on the three fig development phases (Flaishman et al., 2008), we subdivided the fig development process into six stages: stages 1 and 2 belonging to phase I, a rapid growth stage; stages 3 and 4 belonging to phase II, during which fruit size and hardness remain almost unchanged; stages 5 and 6 belonging to phase III, the mature stage, where stage 5 corresponds to commercial ripeness. Inflorescences and receptacles at each stage were separated, marked as F1–F6 and R1–R6, respectively, and stored at −80°C for further RNA-seq and proteomic analyses.

Fig fruit latex was collected by cutting the stage 5 fruit peel with a scalpel; the latex that flowed out was collected into centrifuge tubes and frozen in liquid nitrogen, then stored at −80°C for protein identification.

### RNA-Seq and Quantitative Real-Time PCR (qRT-PCR) Validation

RNA was isolated from samples using the modified CTAB method (Chai et al., 2014). Library construction and RNA-seq methods were as described previously (Wang et al., 2017). The gene-expression profiles during fig development were analyzed based on Illumina RNA sequencing data and annotated against the published fig genome sequence. The transcriptome data of fig fruit at the six development stages were deposited in NCBI (accession number: PRJNA723733). Transcript abundance of fig *PLCP*s was calculated as fragments per kilobase of exon model per million mapped reads (FPKM) and is displayed in heat maps. Significant gene expression was defined by *p*-adjust < 0.05 and | log2(fold change) | ≥ 1.

Transcriptome data of fig fruit after ethylene application (Cui et al., 2020) and under light deprivation (Wang et al., 2019) were re-mined to explore the changes in PLCP gene expression under these treatments. Briefly, fig fruit in phase II were injected with 1 mL of 250 mg/L ethephon solution through the ostiole, RNA-seq was carried out on the inflorescence and receptacle at 2, 4, and 6 days after treatment, and the transcriptome data were stored at NCBI (SRA accession: PRJNA606407) (Cui et al., 2020). For the light-deprivation treatment, fig fruit in stage 2 were covered with opaque paper bags and the transcriptomes of the inflorescence and receptacle of light-deprived and control fruit were determined in commercial-ripe fruit. The complete dataset can be found in the NCBI SRA database (accession number PRJNA494945) (Wang et al., 2019).

The expression of eight PLCP genes was validated by qRT-PCR. PrimeScript^TM^ RT reagent kit (RR037Q, Takara, Dalian, China) was used to reverse transcribe the total RNA. The 18S gene was used for normalization. Primers were designed by Beacon Designer 7 software (Supplementary Table 1). The qPCR was performed with ChamQ Universal SYBR qPCR Master Mix (Q711-02, Vazyme, Nanjing, China). A 15-μL reaction mixture was added to each well. The PCR program was as follows: 95°C for 30 s, and 40 cycles of: 95°C for 10 s, 60°C for 30 s. The 2^–ΔΔ^*^C^*^t^ method (Livak and Schmittgen, 2001) was used for relative quantification analysis with three replicates for each sample.

### Quantitative Proteomic Analysis

Stage 1, 3, and 5 inflorescences and receptacles were used for proteomic analysis. Protein extraction and quantitative analysis were as described in Cui et al. (2019). Three biological replicates were performed for each sample. The digested peptides were labeled with TMT10plex^TM^ Isobaric Label Reagent Set (Thermo Scientific) according to the manufacturer’s instructions. A Q-Exactive mass spectrometer (Thermo Fisher Scientific, Waltham, MA, United States) was used to detect peptide signals. The MS scans were run as described in our previous publication (Cui et al., 2019). The MS results were input into PD software (Proteome Discoverer 1.4, Thermo) to screen the spectra. The selected peptides were identified using Mascot (version 2.3.01) and annotated according to the UniProt database. Then the peptides were quantified by PD software based on their annotation and spectrum. ANOVA was performed to evaluate the significance of the differences. Proteins with a *p*-value less than 0.05, and fold change ≥ 1.2 or ≤0.83 were considered differentially abundant proteins (DAPs). The mass spectrometry proteomics data of inflorescences and receptacles were deposited in the ProteomeXchange Consortium via the PRIDE (Perez-Riverol et al., 2019) partner repository with the dataset identifier PXD025170.

The tris-phenol method (Xie et al., 2009) was used to extract latex protein. A 2-D Quant-kit was used to quantify the protein, and 20 μg protein was applied to SDS-PAGE. The latex protein was excised from the gel and cut into 10 pieces, digested with trypsin enzyme diluted with NH_4_HCO_3_ solution, and the peptides obtained from the digestion were separated by multidimensional liquid chromatography (Dionex Ultimate 3000 nano-LC system), then detected and analyzed by tandem MS (Thermo Fisher Q-Exactive). Mascot (version 2.3.01), MaxQuant (version 1.5.2.8), Thermo Scientific Proteome Discoverer (version 1.3/1.4), and UniProt database were used for protein identification and quantification. The mass spectrometry proteomics data of latex were deposited in the ProteomeXchange Consortium via the PRIDE partner repository with the dataset identifier PXD025485.

## Results

### PLCP Gene Identification and Phylogenetic Analysis

**FIGURE 1:**
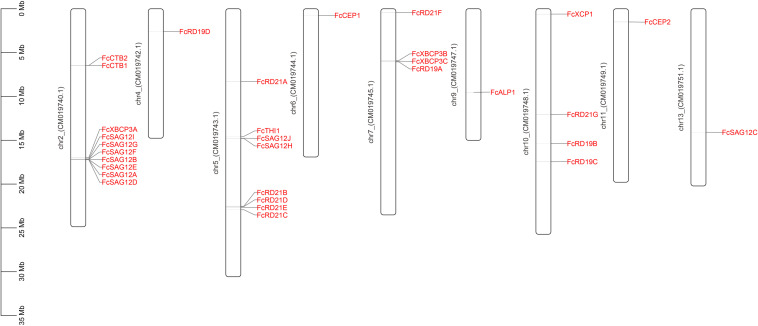
Chromosomal location of 31 FcPLCP genes. The chromosome number is marked on the left side of the chromosome.

Thirty-one PLCP genes were identified in the fig genome by HMM search and manual screening, and mapped to nine chromosomes. Chromosomes 2, 5, 7, and 10 had 10, 8, 4, and 4 *PLCP*s, respectively, and chromosomes 4, 6, 9, 11, and 13 each had 1 *PLCP* (Figure 1).

**FIGURE 2:**
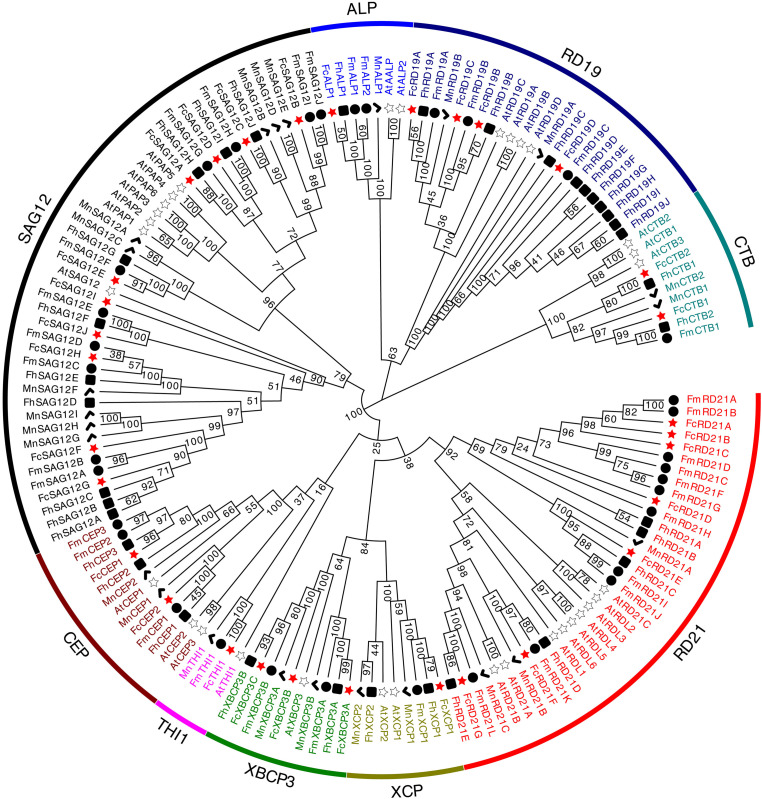
Phylogenetic tree of 156 PLCP genes identified in *Ficus carica*, *Arabidopsis thaliana*, *Morus notabilis*, *Ficus hispida*, and *Ficus microcarpa* genomes. Sequence alignment was performed using ClustalW. The phylogenetic tree was constructed using the neighbor-joining method by MEGA X (1,000 bootstrap).

To investigate the evolutionary relationship among *PLCP*s, we constructed a phylogenetic tree using the fig *PLCP*s together with 32 *PLCPs* from *A. thaliana*, 24 *PLCP*s from *M. notabilis*, 35 *PLCP*s from *F. hispida* and 34 *PLCP*s from *F. microcarpa* (Supplementary Table 2). The *PLCP*s were clustered into nine subfamilies (Figure 2). Based on the homology to *A. thaliana*, we named the nine subfamilies as in Richau et al. (2012), i.e., RD21 (responsive to desiccation 21), CEP (cysteine endopeptidase), XCP (xylem cysteine peptidase), XBCP3 (xylem bark cysteine peptidase 3), THI1 (Th1 immune response-associated cysteine protease), SAG12 (senescence-associated gene 12), RD19 (responsive to desiccation 19), ALP (aleurain-like protease), and CTB (cathepsin B-like). These subfamilies can also be classified according to their closet animal counterparts: cathepsin B (CTB subfamily), cathepsin F (RD19 subfamily), cathepsin H (ALP subfamily), and cathepsin L (SAG12, THI1, CEP, XBCP3, XCP, and RD21 subfamilies) (Martinez and Diaz, 2008; Richau et al., 2012). SAG12 was the largest PLCP subfamily of fig and mulberry, with 10 and 9 members, respectively. RD21, with 7 members, was the second largest subfamily in fig.

### *K*_a_/*K*_s_ Analysis

Genome replication, segmental duplication, and tandem duplication are considered the main evolutionary forces. We identified six duplicated gene pairs in the fig *PLCP* family (Table 1). The *K*_a_/*K*_s_ ratios of *FcRD21A*/*FcRD21B*, *FcRD21A*/*FcRD21C*, *FcRD21B*/*FcRD21C*, and *FcSAG12F*/*FcSAG12G* were <1, suggesting negative selection on these four pairs. The *K*_a_/*K*_s_ ratios of *FcRD19A*/*FcRD19B* and *FcRD19B*/*FcRD19C* were >1, suggesting positive selection for these two pairs. In general, negative selection (*K*_a_/*K*_s_ < 1) eliminates deleterious mutations, retaining the original function of the protein. Positive selection (*K*_a_/*K*_s_ > 1) changes the protein, usually related to coevolution of immune system genes and parasites (Hurst, 2002). Duplication of these six PLCP gene pairs were calculated to have occurred between 1.76 and 59.98 million years ago.

**TABLE 1:** *K*_a_/*K*_s_ calculation and divergence time of the duplicated *FcPLCP* gene pairs.

Duplicated pair	*K*_a_	*K*_s_	*K*_a_/*K*_s_	Divergence time (MYA)
FcRD19A/FcRD19B	0.4155	0.1305	3.1840	9.32
FcRD19B/FcRD19C	1.0683	0.8398	1.2721	59.98
FcRD21A/FcRD21B	0.1051	0.1133	0.9277	8.09
FcRD21A/FcRD21C	0.1087	0.1908	0.5697	13.63
FcRD21B/FcRD21C	0.0840	0.1127	0.7459	8.05
FcSAG12F/FcSAG12G	0.0072	0.0246	0.2933	1.76

*K_a_/K_s_ < 1, negative selection; K_a_/K_s_ = 1, neutral selection; K_a_/K_s_ > 1, positive selection; MYA, million years ago.*

### Gene Structure and Sequence Features

Gene structure analysis showed that the PLCP genes of *F. carica*, *A. thaliana*, and *M. notabilis* differ in intron numbers, whereas the intron numbers within a subfamily were conserved (Supplementary Figure 1). Subfamilies RD21 and RD19 featured 2–5 introns and 2–4 introns, respectively. The other subfamilies had 1–10 introns. The full lengths of the *FcPCLP*s were from 1,128 to 5,075 bp with CDSs ranging from 624 to 1,518 bp.

**FIGURE 3:**
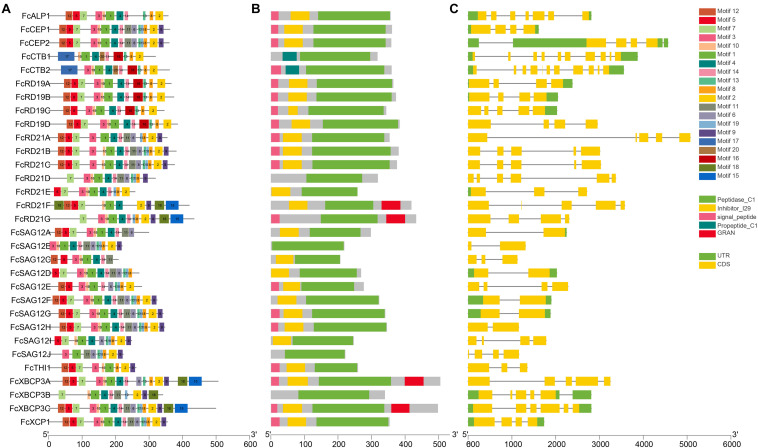
Analysis of gene structure and conserved motifs of *FcPLCP*s. **(A)** Distribution of 20 conserved motifs of FcPLCP proteins. The conserved motifs are represented by different numbers and block colors. **(B)** Distribution of primary domains and signal peptides on FcPLCP proteins. **(C)** Graphic representation of exon–intron structures.

Multiple sequence alignment revealed conserved motifs in the *FcPLCP*s (Table 2 and Supplementary Figure 2); most *FcPLCP*s in the same subfamily had similar motifs, indicating that the protein structures were conserved (Figure 3). Motifs 5, 7, and 12 were identified as the inhibitor I29 domain (pfam08246), acting as propeptides that can inhibit protease activity. Most of the *FcPLCP*s had the inhibitor I29 domain, which contains the ERFNIN motif (Richau et al., 2012)—an interspersed amino acid motif in the N-terminal propeptide region with a highly conserved EX_3_RX_2_(V/I)FX_2_NX_2_IX_3_N sequence. RD19 subfamily *FcPLCP*s carried a conserved ERFNAQ motif instead of ERFNIN. In the CTB subfamily, motif 17, characterized as a propeptide, replaced the inhibitor I29 domain at the N terminus.

**TABLE 2:**
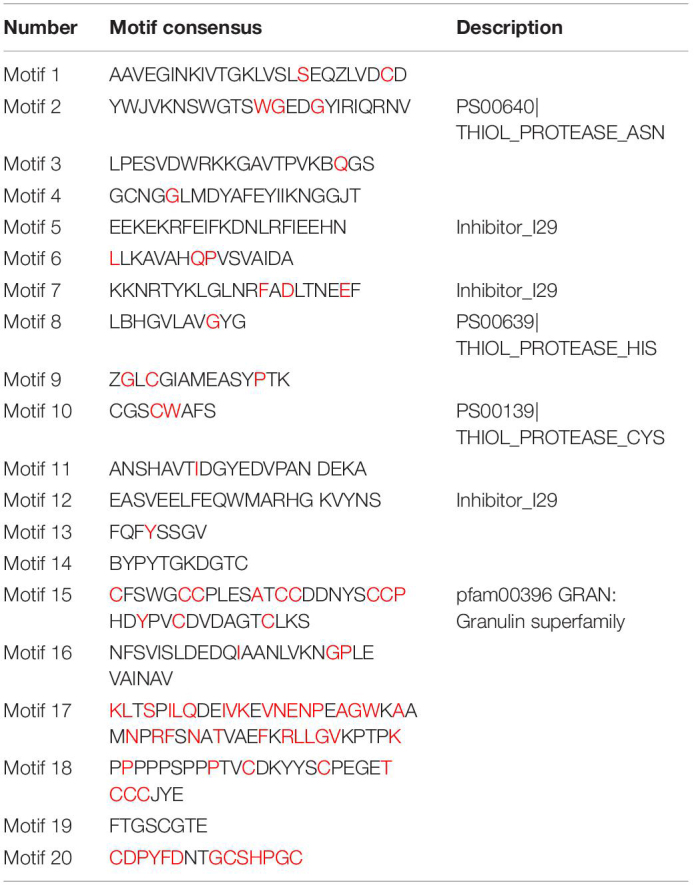
Putative motifs of fig PLCPs identified by MEME program.

*The conserved motifs exported by MEME program using the PLCP sequences of F. carica, A. thaliana, and M. notabilis. Letters in red are conserved residues.*

Motifs 1, 2, 3, 4, 6, 8, 9, 10, 11, 13, 14, and 19 were identified as belonging to the peptidase C1 domain (PF00112). Motifs 2, 10, and 8 had Asn, Cys, and His catalytic sites, respectively. Motif 10 of some SAG12 subfamily members had a double Cys in the catalytic site. The distribution of the specific conserved motifs varied among subfamilies. In RD19, ALP, and CTB subfamilies, motif 16 replaced motifs 11 and 6. In the RD21 and XBCP3 subfamilies, motifs 18 and 15 were usually located at the C terminus (Figure 3).

Motif 15 appeared in two members of the RD21 subfamily and two members of the XBCP3 subfamily (Figure 3). It was identified as a granulin domain (PF00396). In plants, the granulin motif has been found at the C terminus of some cysteine proteases whose expression is upregulated under environmental stress (Bateman and Bennett, 2009). Not every XBCP3 and RD21 subfamily member carried the granulin motif, suggesting that the granulin polymorphism evolved by domain loss (Richau et al., 2012).

All CEP subfamily *FcPLCP*s had the KDEL sequence at the C terminus for retention in the endoplasmic reticulum. Than et al. (2004) found that removing the N-terminal propeptide and C-terminal KDEL sequence under acidic conditions results in ricinosome maturation. We also found that the N terminus of *FcALP1* carries an NPIR signal known as a vacuolar-targeting sequence.

Fig PLCPs are 207 to 505 amino acids in length, and have a molecular mass of 23.09–56.19 kDa and pI of 3.88 to 9.14 (Supplementary Table 2). The GRAVY index of FcPLCPs was from −0.59 to −0.14, indicating they are hydrophilic; 19 FcPLCPs, 31 AtPLCPs, and 20 MnPLCPs were predicted to carry N-terminal signal peptides (Figure 3). In several PLCP sequences annotated in the present study, neither signal peptides nor propeptides were identified. This could be due to the assembly quality of the used fig genome (Usai et al., 2020, 74-fold coverage of the cv. Dottato haploid genome) and the possible existence of pseudogenes. Most FcPLCPs were predicted to be soluble proteins. Subcellular localization analysis showed that 24, 5, and 2 FcPLCPs were located in the lysosome/vacuole, extracellular matrix and cytoplasm, respectively. All 32 AtPLCPs were predicted to be localized to the lysosome/vacuole. MnPLCPs were located in the lysosome/vacuole (21 PLCPs), extracellular matrix (1 PLCP), plastid (1 PLCP), and cytoplasm (1 PLCP) (Supplementary Table 3).

### Promoter Analysis

**FIGURE 4:**
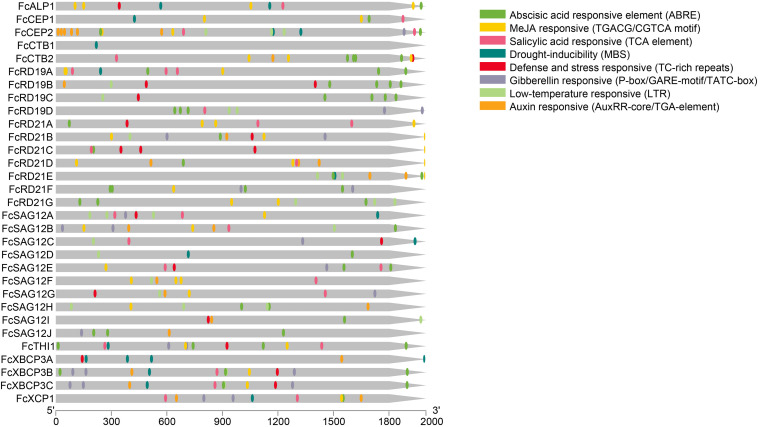
Promoter analysis of *FcPLCP*s by Plant CARE, based on 2,000-bp sequence upstream of the genes. Detailed information on *cis*-elements is given in Supplementary Table 4.

All *FcPLCP* promoters contained at least one putative biotic/abiotic stress response element; 25 *PLCP*s contained an abscisic acid response element, 22 contained a methyl jasmonate response element, 19 contained a salicylic acid response element, 17 contained a low-temperature response element, 16 contained an auxin response element, 14 had a gibberellin response element, 16 had a defense and stress response element and 14 had a drought response element (Figure 4), suggesting that *FcPLCP*s are involved in stress responses. Moreover, all of the *FcPLCP* promoters contained light response elements, and 29 of them contained anaerobic induction elements, indicating that their expression is regulated by light and oxygen (Supplementary Table 4).

### Expression Pattern of *PLCP*s in Fig Fruit Development

**FIGURE 5:**
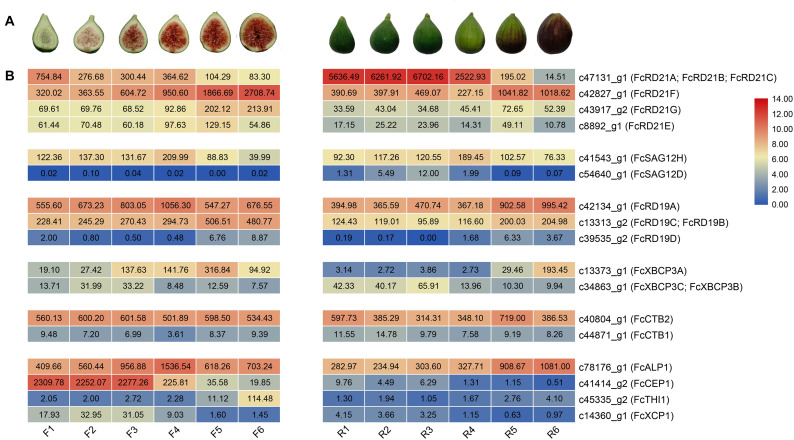
*FcPLCP* expression during fig fruit development. **(A)** Images represent six developmental stages of inflorescence (F1–F6) and receptacles (R1–R6). **(B)** Numbers represent FPKM values. Color scale represents FPKM normalized log2-transformed counts. Blue color, low expression; red color, high expression. *FcPLCP*s are grouped according to their subgroup.

Seventeen *PCLPs* were identified in the inflorescence and receptacle transcriptomes of the six fig fruit developmental stages; no tissue-specific *PCLP* was found in the inflorescence or receptacle. The RD21 subfamily made up the largest proportion of detected expressed *PCLP*s (Figure 5).

Along inflorescence development, the expression of *c41414_g2* (*FcCEP1*) and *c47131_g1* (*FcRD21A*, *FcRD21B*, *FcRD21C*) decreased. In contrast, the expression of *c42827_g1* (*FcRD21F*), *c13313_g2* (*FcRD19C*, *FcRD19B*), *c13373_g1* (*FcXBCP3A*), *c43917_g2* (*FcRD21G*), and *c45335_g2* (*FcTHI1*) increased. The expression of *c42134_g1* (*FcRD19A*) and *c78176_g1* (*FcALP1*) first increased and then decreased; *c41414_g2* (*FcCEP1*) expression was abundant at the early stage.

PLCP genes *c42827_g1* (*FcRD21F*), *c42134_g1* (*FcRD19A*), *c78176_g1* (*FcALP1*), *c13313_g2* (*FcRD19C*, *FcRD19B*), and *c13373_g1* (*FcXBCP3A*) were upregulated at the late stage of receptacle development, whereas *c47131_g1* (*FcRD21A*, *FcRD21B*, *FcRD21C*), annotated as ficin, was highly expressed from R1 to R4, and then dropped in R5 and R6. The expression of *c78176_g1* (*FcALP1*), which was also annotated as ficin, increased during receptacle development.

The changes in transcription abundance of eight *PLCP*s during fig fruit development were verified by qRT-PCR. The expression trends were consistent with the RNA-seq results, indicating their reliability (Supplementary Figure 3). The quality of the RNA-seq of fig fruit after ethylene treatment and under light deprivation were validated by qRT-PCR when the data were analyzed for the original purposes of the studies (Wang et al., 2019; Cui et al., 2020).

### Change in Abundance of PLCP Proteins During Fig Fruit Development

Protein identification and quantification were performed at three developmental stages (stages 1, 3, and 5) for inflorescences and receptacles. Eighteen PLCPs were annotated, and 13 of them were identified as DAPs. Their fold changes are shown in Table 3 and Supplementary Table 5.

**TABLE 3:**
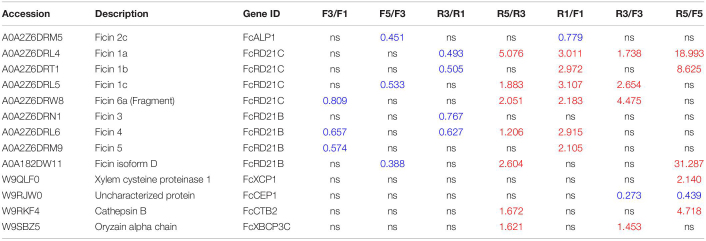
Papain-like cysteine proteases (PLCPs) identified in fig fruit soluble proteome and comparison of their abundance in the inflorescence and receptacle during fruit development.

*Proteins with p < 0.05 and fold change ≥ 1.2 or ≤0.83 were identified as differentially abundant. ns, no significant difference in abundance; F, inflorescence; R, receptacle; 1, 3, and 5 represent fruit development stages corresponding to phase I (early stage of fruit development), late phase II (before fruit begins to ripen) and middle of phase III (commercial-ripe), respectively. Blue values represent downregulated; red values represent upregulated.*

Through protein sequence alignment, ficin 2C was identified as FcALP1, ficin 1A, ficin 1B, and ficin 1C were FcRD21C, and ficin 3 was identified as FcRD21B. Moreover, ficin D, ficin 4, and ficin 5 exhibited 72.15, 74.54, and 78.13% sequence identity with FcRD21B, respectively, and ficin 6A had 78.33% sequence identity with FcRD21C (Supplementary Figure 4). Along fruit development, the abundance of ficins decreased in the inflorescence, e.g., ficin 2C was a downregulated DAP in F5 compared to F3 (F5/F3). In the receptacle, the abundance of these ficins decreased in stage 3 but increased in stage 5. Aside from ficin 2C (FcALP1), most ficin proteins were of higher abundance in the receptacle than in the inflorescence.

In addition to ficins, some other PLCPs were identified as DAPs, including FcXCP1 (increased in R5/F5), FcCTB2 (increased in R5/F5 and R5/R3), FcXBCP3C (increased in R5/R3), and FcCEP1 (decreased in R3/F3 and R5/F5).

### PLCP Proteins in Fig Fruit Latex

Seventy-four proteins were identified in the latex, with molecular masses ranging from 8.9 to 206.1 kDa and pI values of 4.84 to 10.78. Latex contained many stress-response proteins, including trypsin-like protease inhibitor, chitinase, endochitinase, and pathogenesis-related (PR) protein isoforms, indicating that it plays an essential role in resistance to insects and microbes. PLCPs were the most abundant protein component in fig latex at the commercial-ripe stage, accounting for 38.93% of the total protein content. The identified PLCPs included ficin 4 (14.93%), ficin 1B (8.63%), ficin 1A (8.04%), W9RY43 (5.82%, FcRD21G), and ficin D (1.50%) (Table 4 and Supplementary Table 6).

**TABLE 4:** Papain-like cysteine proteases identified in the commercial-ripe fig fruit receptacle latex proteome.

Accession	Description	Score	Coverage (%)	PSMs	Intensity	Relative amount (%)
A0A2Z6DRL6	Ficin 4	1949.16	99.54	2680	145000000	14.93
A0A2Z6DRT1	Ficin 1b	1477.69	83.33	2389	83800000	8.63
A0A2Z6DRL4	Ficin 1a	1606.25	90.50	2169	78100000	8.04
A0A182DW11	Ficin isoform D	230.57	16.36	287	14600000	1.50
W9RY43	Cysteine proteinase RD21a	186.08	8.19	72	56500000	5.82

*Detailed information can be found in Supplementary Table 6. PSMs, the peptide-spectrum matches.*

### Expression Pattern of *PLCPs* in Fig Fruit Treated With Ethephon and Light Deprivation

**FIGURE 6:**
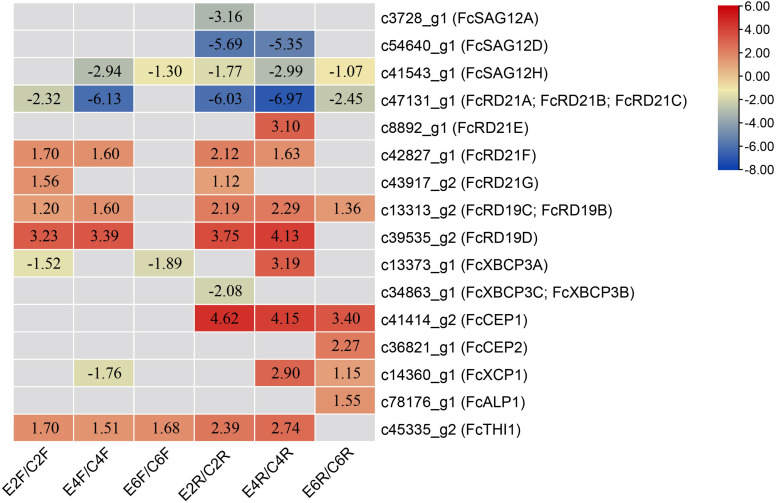
Differentially expressed PLCP genes upon ethephon treatment. Genes were defined as differentially expressed by *p*-adjust < 0.05 and | log2(fold change)| ≥ 1. Numbers represent log2(fold change). Inflorescences treated with ethephon for 2, 4, and 6 days: E2F, E4F, and E6F, respectively; receptacles treated with ethephon for 2, 4, and 6 days: E2R, E4R, and E6R, respectively. Control inflorescence and receptacles on days 2, 4, and 6: C2F, C4F, C6F, C2R, C4R, and C6R, respectively.

Phase II fig fruit were treated with 1 mL of 250 mg/L ethephon (Cui et al., 2020). The RNA-seq data for the inflorescence and receptacle at 0, 2, 4, and 6 days after treatment were analyzed. Sixteen *PLCP*s showed differential expression (Figure 6). Eleven *PLCP*s were upregulated in the inflorescence or receptacle; among them, *c39535_g2* (*FcRD19D*), *c43917_g2* (*FcRD21G*), *c42827_g1* (*FcRD21F*), *c45335_g2* (*FcTHI1*), *c13313_g2* (*FcRD19C*, *FcRD19B*) were significantly upregulated in both the inflorescence and receptacle. Seven *PLCP*s were downregulated in the inflorescence or receptacle after ethephon treatment. Among them, *c47131_g1* (*FcRD21A*, *FcRD21B*, *FcRD21C*) was the most downregulated transcript.

Fig fruit were deprived of light by bagging at stage 2 of development to study the biology of fig color development (Wang et al., 2019). The RNA-seq data for the inflorescences and receptacles of ripe fruit revealed 3 and 4 differentially expressed *PLCP*s, respectively: all of them were downregulated under light deprivation (Table 5). Genes *c47131_g1* (*FcRD21A*, *FcRD21B*, and *FcRD21C*) and *c8892_g1* (*FcRD21E*) were repressed in both the inflorescence and receptacle, whereas *c56852_g1* (*FcSAG12F*, *FcSAG12G*) was only downregulated in the inflorescence, and *c39535_g2* (*FcRD19D*) and *c13373_g1* (*FcXBCP3A*) were only downregulated in the receptacle.

**TABLE 5:** Differentially expressed PLCP genes in fig fruit subjected to light deprivation.

Gene ID	Inflorescence control (FC) (FPKM)	Inflorescence light deprivation (FL) (FPKM)	log2FC(FL/FC)	Receptacle control (RC) (FPKM)	Receptacle light deprivation (RL) (FPKM)	log2FC (RL/RC)
c56852_g1 (FcSAG12F; FcSAG12G)	4.98	0.41	−3.62	ns	ns	ns
c47131_g1 (FcRD21A; FcRD21B; FcRD21C)	174.26	31.18	−2.48	66.76	15.60	−2.09
c8892_g1 (FcRD21E)	18.58	5.67	−1.71	17.19	4.38	−1.97
c39535_g2 (FcRD19D)	ns	ns	ns	10.08	2.27	−2.15
c13373_g1 (FcXBCP3A)	ns	ns	ns	55.83	5.93	−3.23

*ns, no significant difference; Log2FC (fold change).*

## Discussion

### PLCPs in *Ficus* Species

Fig belongs to the genus *Ficus* in the family Moraceae. The genus has about 700 species, most of them evergreens, including trees, shrubs and climbers growing under different climatic conditions (Flaishman et al., 2008; Zhang et al., 2020). In three sequenced *Ficus* species genomes, 31, 35 and 34 PLCP genes were identified (*F. carica*, *F. hispida* and *F. microcarpa*, respectively). The number of PLCPs varied among species and subfamilies, possibly due to whole-genome duplication, tandem duplication, and large-scale segmental duplication (Liu et al., 2018). In eukaryotic species, gene duplications are estimated to occur at an average rate of 0.01 per gene per million years (Lynch and Conery, 2000).

*PLCP*s of the three *Ficus* species differed in the structure of their subfamilies. In *F. carica*, SAG12 (10 members) and RD21 (7) were the two largest subfamilies, whereas in *F. hispida*, SAG12 (10) and RD19 (10) were the two largest; the RD21 subfamily had 5 *PLCP*s, and no THI subfamily members were identified, possibly being lost during evolution: the THI subfamily has been reported as lost in poplar (Zou et al., 2017). In *F. microcarpa*, RD21 (11) and SAG12 (10) were the two largest subfamilies. The three *Ficus* species all had 10 SAG12 subfamily members, whereas in mulberry and *Arabidopsis*, SAG12 had 9 and 6 members, respectively. It is speculated that SAG12 *PLCP*s may have formed before the formation of the individual species. SAG12 *PLCP*s are senescence-associated genes and their expression increases in senescing leaves (James et al., 2018).

The RD19 subfamily showed significant expansion in *F. hispida*, and the RD21 subfamily showed significant expansion in *F. microcarpa*, which could be due to local gene duplication. Both RD19 and RD21 subfamilies are involved in disease resistance. *Arabidopsis rd19*-null mutants have been found to be more susceptible than the wild type to the bacterial pathogen *Ralstonia solanacearum*, whereas *rd21*-null mutants show impaired resistance to *Botrytis cinerea* (Misas-Villamil et al., 2016).

In *F. hispida*, 35 *PLCP*s were distributed on 10 of the 14 chromosomes (Supplementary Figure 5); 7 SAG12 subfamily members and 8 RD19 members formed gene clusters on chr1 (GWHALOG00000001) and chr4 (GWHALOG00000004), respectively. The formation of gene clusters may be related to the genes’ tandem duplication. In *F. microcarpa*, 34 *PLCP*s were distributed on 9 of 13 chromosomes (Supplementary Figure 6); 7 SAG12 subfamily members and 9 RD21 members formed gene clusters on chr1 (GWHABKV00000001.1) and chr5 (GWHABKV00000005.1), respectively. In *F. carica*, 31 *PCLP*s were distributed on 9 of 13 chromosomes. Seven SAG12 members and 4 RD21 members formed gene clusters on chr2 (CM019740.1) and chr5 (CM019743.1), respectively. The genome distribution and similar clustering of the PLCP genes from the three *Ficus* species indicated mutual whole-genome duplications and species-specific tandem duplications of specific subfamilies, as reported in castor bean, physic nut (Zou et al., 2018) and cotton (Zhang et al., 2019).

**FIGURE 7:**
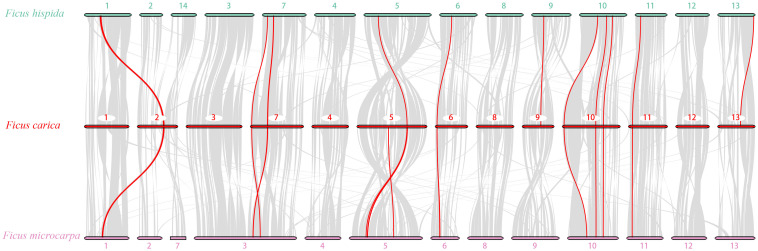
Genome synteny analysis of *F. carica* with *F. hispida* and *F. microcarpa*. Gray lines in the background indicate the collinear blocks among *F. carica* and *F. hispida* and *F. microcarpa*; red lines highlight syntenic *PLCP* pairs.

To further analyze the *PLCP* family’s phylogenetic mechanism, we constructed comparative syntenic maps of *F. carica* associated with *F. hispida* and *F. microcarpa*. The three *Ficus* species’ genomes showed high homology (Figure 7). A total of 16 and 20 *FcPLCP*s showed syntenic relationships with those in *F. hispida* and *F. microcarpa*, respectively (Supplementary Table 7), indicating that *F. carica* may have a closer evolutionary relationship with *F. microcarpa* than with *F. hispida*. Some syntenic gene pairs were conserved in the three *Ficus* species, including some SAG12 and RD21 subfamily members, indicating that their expansion occurred before the three species’ divergence. A chromosome fusion or fission event occurred between *F. microcarpa* chr3 and its homologs, chr3 and chr7 of *F. carica* and *F. hispida*, respectively (Zhang et al., 2020). *FcRD19A*, *FcXBCP3C*, and *FcRD21F* on chr7 were identified as syntenic genes. *F. carica* chr2, *F. microcarpa* chr2 and 7, and *F. hispida* chr2 and 14 may also have undergone fusion or fission events. Several inversions occurred in the three genomes’ chromosomal fragments, calling for further study.

### PLCP Expression Patterns

*Ficus* is characterized by unique aggregate fruit that develop from an enclosed urn-shaped inflorescence; the receptacle serves as a physical barrier, protecting the enclosed inflorescence and small drupelets from disease and insects. A few recent studies have revealed that the inflorescence and surrounding receptacle of figs differ with respect to ripening (Freiman et al., 2015), and their response to gibberellin (Chai et al., 2018, 2019) and ethephon treatment (Cui et al., 2020).

In the present study, among the 31 sequences recruited as PLCP-encoding genes from the published fig genome, 17 *PLCP* transcripts and 18 PLCP proteins were identified, and their spatiotemporal expression/abundance pattern in the inflorescence and receptacle was revealed by transcriptome and proteome analysis, respectively. Limited by the fact that only fig fruit material was used in our study, we are not in a position to suggest which remaining sequences are transcribed/translated in other tissues or, in other words, are pseudogenes. The present results emphasize the divergent roles of the inflorescence and receptacle in fig fruit reproductive biology. The spatial expression pattern of *PLCP*s has been reported in *Arabidopsis* (Richau et al., 2012), rubber tree (Zou et al., 2017), cotton (Zhang et al., 2019), castor bean and physic nut (Zou et al., 2018).

Pollination of the *Ficus* inflorescence relies on species-specific wasps (*Blastophaga psenes* L.) (Flaishman et al., 2008; Zhang et al., 2020). The wasps enter the enclosed fig fruit through the scale-covered ostiole, and pollination occurs inside the urn-shaped syconia at the end of phase I of fig fruit development. Bacterial and fungal pathogens and the diseases that they transmit have been reported in fig production: *Alternaria* and *Fusarium* were the two major pathogens, introduced by fig wasps and producing internal fig rot (Stover et al., 2007). Transcriptome analyses of the inflorescence demonstrated high *CEP1* levels in the young fruit. AtCEP1 has been reported to be essential in tapetum programmed cell death and pollen development (Liu et al., 2018). The relatively high expression of members of the *FcRD21* and *FcRD19* subfamilies, both involved in resistance to biotic stress, suggests that tissue-specific *PLCP* expression could play a role in supporting fig inflorescences’ control of the biological risk associated with the wasp pollinator entering the syconium at the early stage of fruit development.

Among of the 18 PLCPs identified in the fig’s soluble proteome, 6 were identified as DAPs in the inflorescence, all of them ficins. Significantly decreased abundance (*p* < 0.05, fold change ≤ 0.83) was found for ficin 1C, ficin 2C, ficin 4, ficin 5, ficin 6A and ficin isoform D during inflorescence development. When the fig fruit ripens, the decreased abundance of ficin and other PLCPs in the inflorescence—the major edible part of fig fruit—may indicate a decreased requirement for antibacterial agents, thereby facilitating the feeding of dispersing organisms.

A large number of PLCPs, especially ficins, are toxic to herbivorous insects (Konno et al., 2004). The biotic risk faced by the fig receptacle is different from that faced by the inflorescence, the latter being well protected from most insects by the receptacle and scales covering the ostiole. PLCPs of subfamilies RD21, RD19, ALP1, and CTB2 showed high transcription levels in the receptacle. In papaya fruit, subfamily III PLCP genes, including the papain gene *CpXCP5*, are expressed at high levels in stage I fruit; the papain and other major PLCPs in papaya latex provide defense against herbivorous insects as the papaya fruit develops (Liu et al., 2018). In our study, only one putative *FcXCP1* transcript was identified in fig fruit and it showed basal expression in the receptacle, whereas one RD21 member (*c47131_g1*), previously shown to have roles in plant immunity and resistance to necrotrophic fungal pathogens and arthropod crustaceans (Rustgi et al., 2018), exhibited extremely high expression in the receptacle before fig fruit ripening.

Our fig fruit receptacle proteomic data also supported a significant increase in abundance of ficin 4, ficin 1A, ficin 1C, ficin 6A, ficin isoform D, CTB2 and XBCP3C from mid-stage fig development to near commercial ripeness. Latex collected from the receptacle of commercial-ripe figs was rich in ficin 4, ficin 1A, ficin 1B and ficin isoform D. The major PLCP components in the receptacle latex were similar to those found in the receptacle. The presence of ficins in the latex confirms their role in plant resistance to microbes and herbivores (Kitajima et al., 2018). High transcription and protein abundance of the major PLCPs in the fig receptacle in the commercial-ripe fruit suggest strong and persistent PLCP protection of the receptacle against biotic stresses.

Moreover, *PLCP* expression has been reported to be modulated by plant hormones and environmental stimuli. Ethephon is regularly applied to rubber trees to increase the yield of rubber latex (Zhu and Zhang, 2009). In our study, most of the PLCPs were upregulated following ethephon application. Commonly found light-responsive elements in *PLCP* promoters and a comparison of light-deprived and natural grown fig fruit transcriptomes strongly suggest that light is a positive signal in the expression of most *PLCP*s. In support of this, a study with smyrna-type fig cultivars found the highest protease activity in the late afternoon after long light exposure (Lazreg-Aref et al., 2018). Recently, differently changing patterns of protease activity in different fig types and cultivars have been reported. In the common-type cultivar Kahli and the San Pedro-type cultivar Bither Abiadh, protease activity decreases with maturity, whereas in the smyrna-type cultivars Njali and Temri, and the caprifig cv. Besbessi, protease activity increases with maturity (Lazreg-Aref et al., 2018). This shows that in addition to the stage of development, cultivar may also be an important factor affecting *PLCP* expression in fig fruit, warranting further study.

## Conclusion

In this study, the PLCP family was analyzed in fruit of the edible fig *F. carica* at the level of gene structure, sequence characteristics, promoter *cis*-elements, expression patterns and proteomics. Species-specific PLCP subfamily duplication was revealed, which could be relevant to the uniqueness of edible fig, being the only deciduous species of *Ficus*, which has been under long selection pressure by humans as one of the earliest domesticated fruit trees. High expression of disease- and herbivore-resistance/repelling PLCP genes and a high abundance of ficins in the inflorescence, receptacle and fruit latex provide valuable information on fig fruit developmental biology. Comparisons with other *Ficus* species and a comparison between cultivated figs (common fig) and wild-type figs (caprifig) are needed to better understand *Ficus* PLCPs and their role in plant development, and especially in biotic stress responses.

## Data Availability Statement

The datasets generated for this study can be found in online repositories. The names of the repository/repositories and accession number(s) can be found below: NCBI, accession numbers: PRJNA606407, PRJNA494945, PRJNA723733, and ProteomeXchange consortium, via PRIDE partner repository: PXD025170 and PXD025485.

## Author Contributions

YZ, YC, and MS conducted the experiments and data analyses. YZ, AV, SC, and HM prepared the manuscript. All authors have read and approved the manuscript for publication.

## Conflict of Interest

The authors declare that the research was conducted in the absence of any commercial or financial relationships that could be construed as a potential conflict of interest.

## Footnotes

1 https://www.ncbi.nlm.nih.gov/

2 https://www.arabidopsis.org/

3 https://bigd.big.ac.cn/gsa/

4 https://www.ncbi.nlm.nih.gov/Traces/wgs/VYVB01?display=history%EF%BC% 89

5 https://www.ncbi.nlm.nih.gov/cdd/

6 http://web.expasy.org/protparam/

7 https://services.healthtech.dtu.dk/service.php?SignalP-5.0

8 https://services.healthtech.dtu.dk/service.php?DeepLoc-1.0

## Supplementary Material

Supplementary Figure 1 Analysis of gene structure and conserved motifs of *Ficus carica, Arabidopsis thaliana*, and *Morus notabilis.*

Supplementary Figure 2 Conserved motifs of fig PLCPs identified by MEME program. Letters represent amino acids, their size represents the conservation of residues at individual positions.

Supplementary Figure 3 Transcriptomic changes during fig fruit development validated by qRT-PCR using 8 PLCP genes.

Supplementary Figure 4 Sequence alignment of FcPLCPs and previously identified ficins.

Supplementary Figure 5 Chromosomal location of *Ficus hispida* PLCP genes.

Supplementary Figure 6 Chromosomal location of *Ficus microcarpa* PLCP genes.

Supplementary Table 1 Primer sequences of 8 FcPLCP genes used for quantitative real-time PCR.

Supplementary Table 2 Papain-like cysteine proteases (PLCP) gene and predicted protein features of *Arabidopsis thaliana*, *Morus notabilis*, *Ficus hispida* and *Ficus microcarpa.*

Supplementary Table 3 Prediction of FcPLCPs’ subcellular location.

Supplementary Table 4 *Cis*-element analysis of *FcPLCP* promoters (2000 bp).

Supplementary Table 5 Identification and relative quantification of PLCPs in fig soluble proteome.

Supplementary Table 6 Proteins identified from commercial-ripe fig fruit receptacle latex proteome.

Supplementary Table 7 Syntenic gene pairs within *F. carica*, *F. hispida*, and *F. microcarpa*.
