# Supplementary figures and images for: Papain-Like Cysteine Protease Gene Family in Fig (Ficus carica L.): Genome-Wide Analysis and Expression Patterns
Source: Front Plant Sci. 2021 May 28;12:681801. doi: 10.3389/fpls.2021.681801 (PMC8193581; doi:10.3389/fpls.2021.681801)

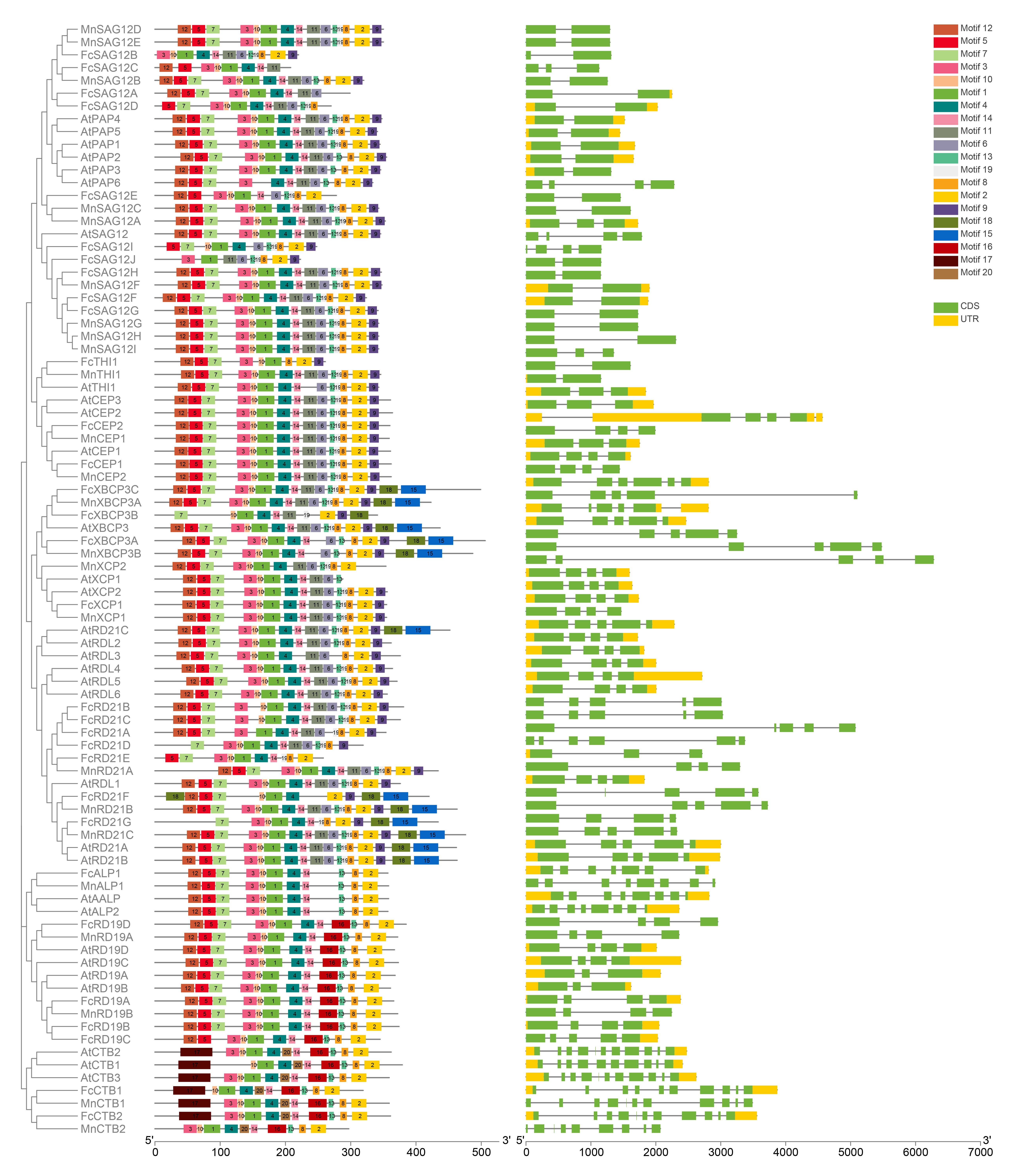

Supplement: Supplementary Figure 1 — Analysis of gene structure and conserved motifs of Ficus carica, Arabidopsis thaliana, and Morus notabilis. [file Image_1.JPEG]

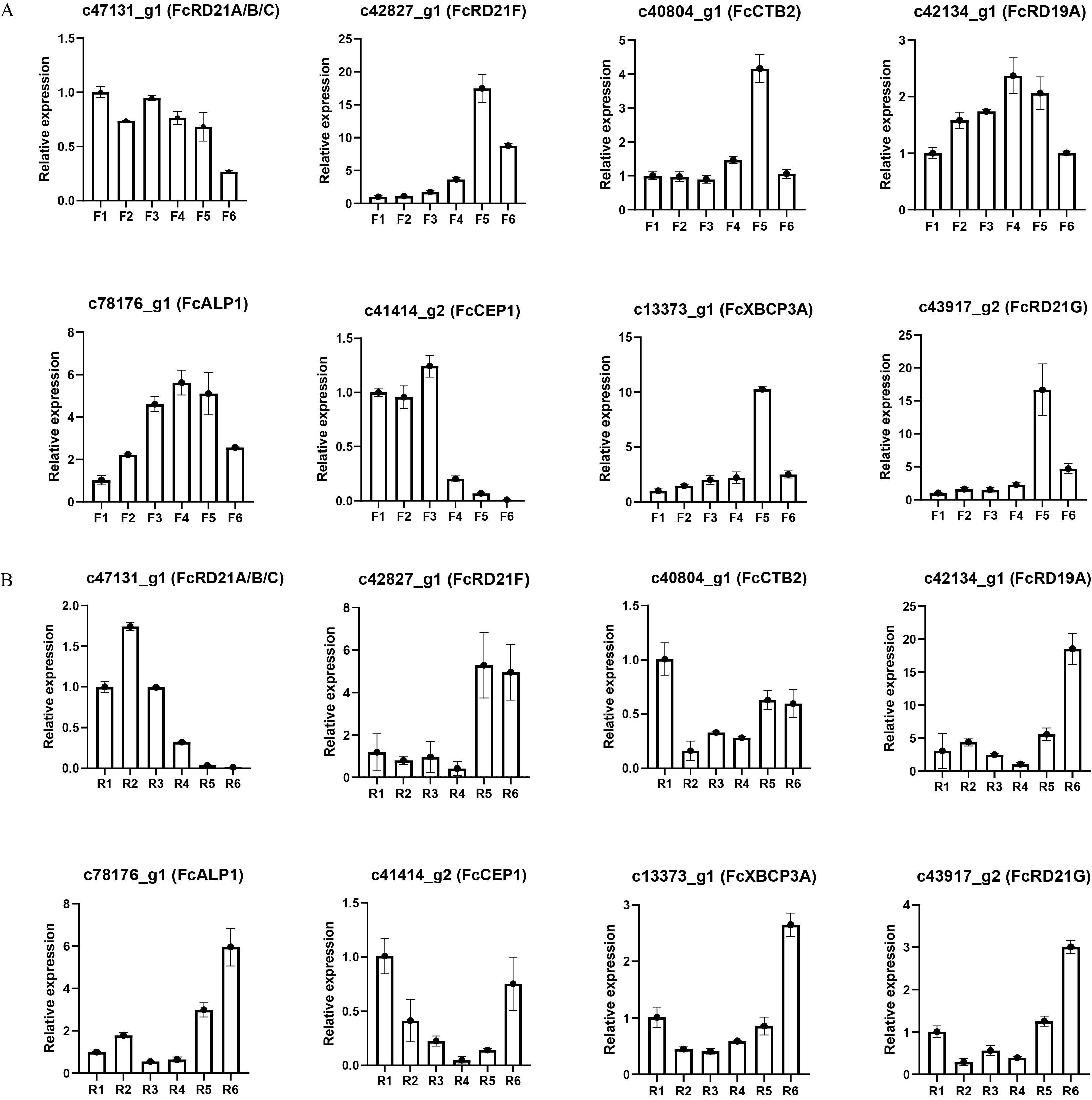

Supplement: Supplementary Figure 3 — Transcriptomic changes during fig fruit development validated by qRT-PCR using 8 PLCP genes. [file Image_3.JPEG]

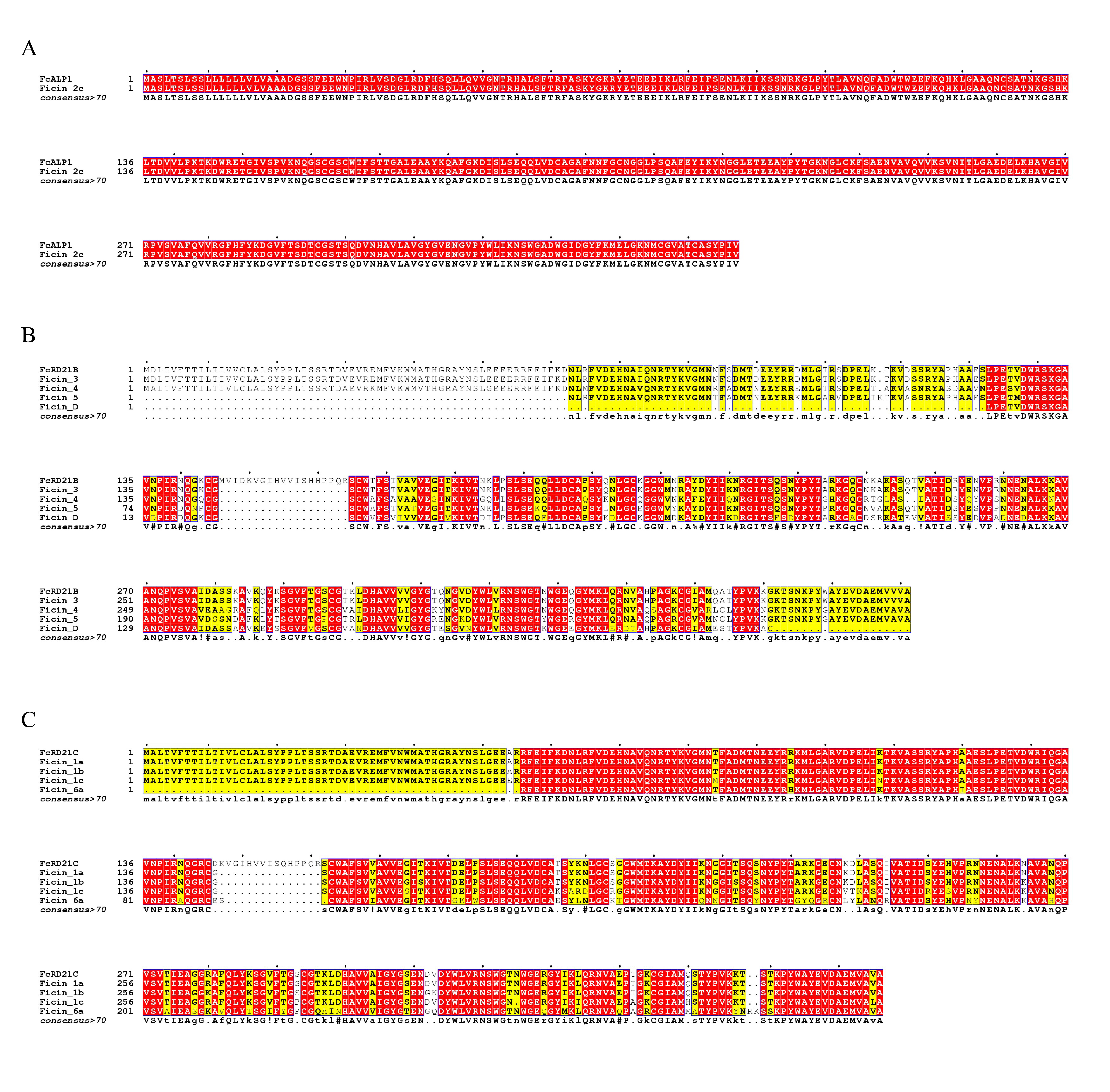

Supplement: Supplementary Figure 4 — Sequence alignment of FcPLCPs and previously identified ficins. [file Image_4.JPEG]

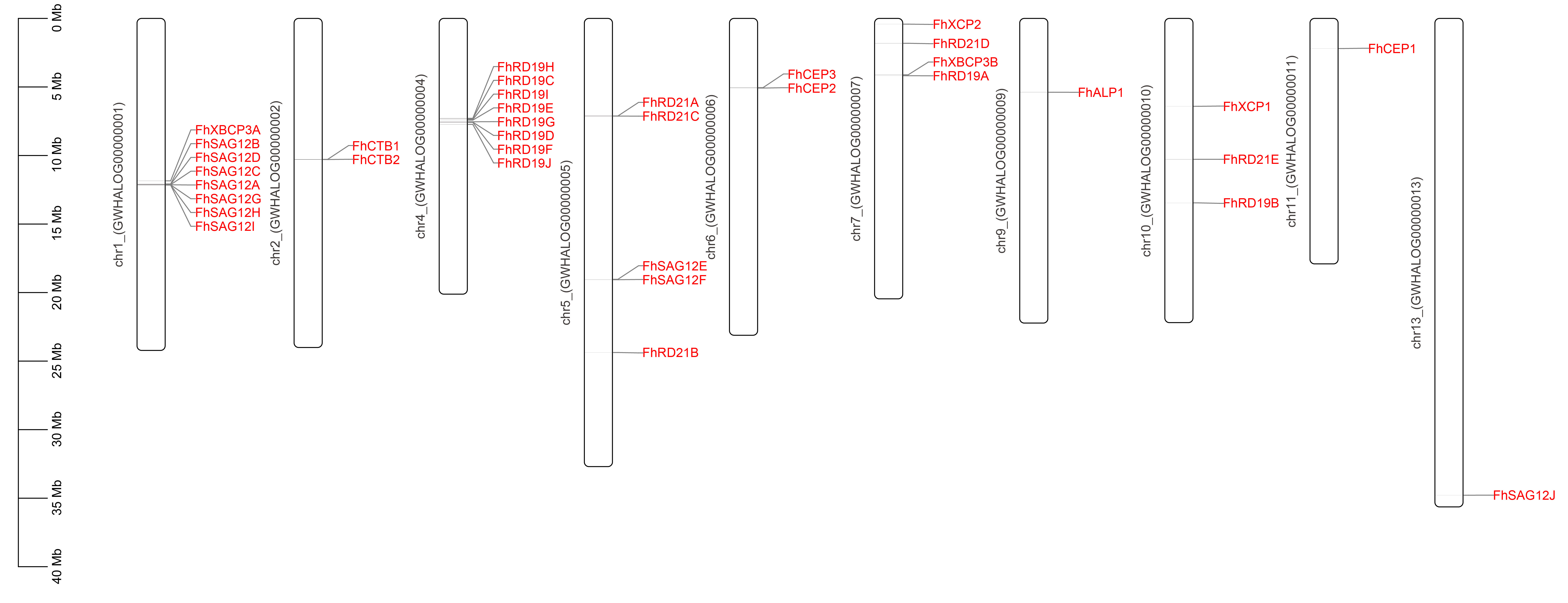

Supplement: Supplementary Figure 5 — Chromosomal location of Ficus hispida PLCP genes. [file Image_5.JPEG]

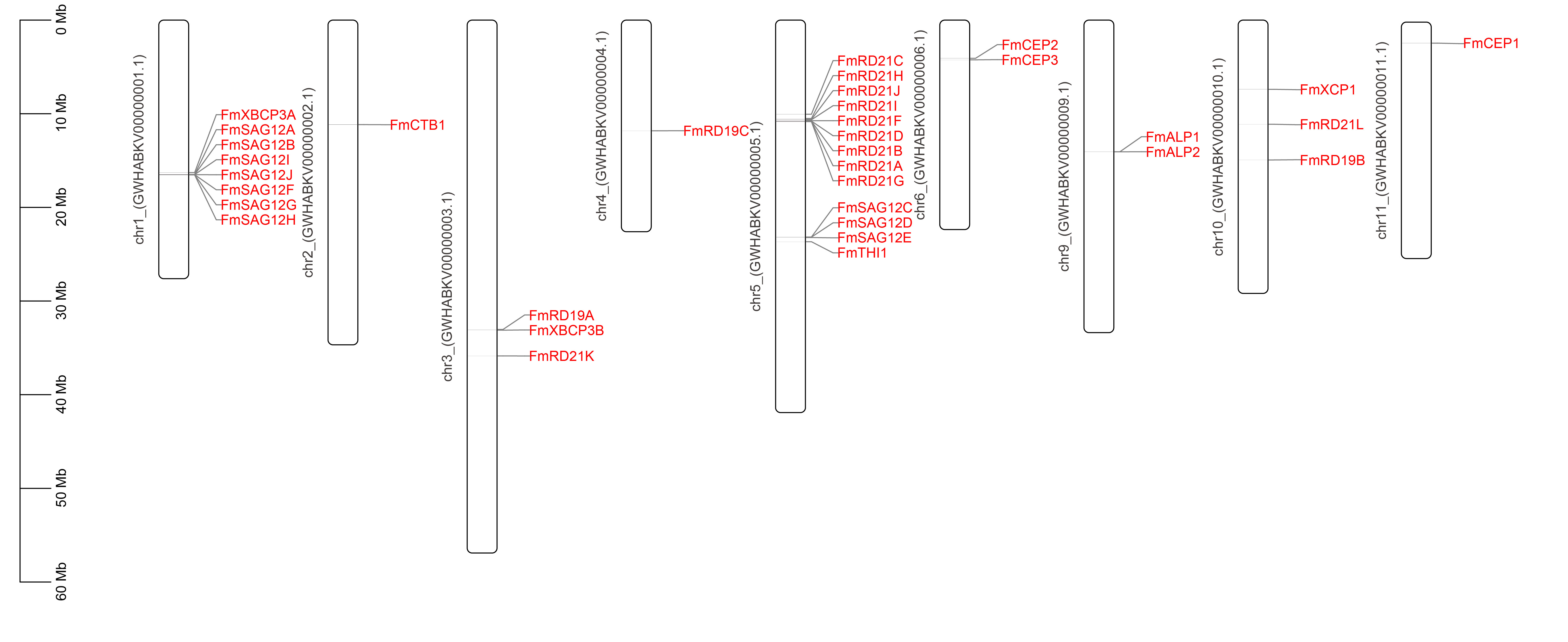

Supplement: Supplementary Figure 6 — Chromosomal location of Ficus microcarpa PLCP genes. [file Image_6.JPEG]
